# Non-pharmacological therapies for breast cancer-related lymphedema: a systematic review and network meta-analysis based on randomized controlled trials

**DOI:** 10.3389/fonc.2026.1828957

**Published:** 2026-05-29

**Authors:** Ningning Fang, Hong Xin, Mengmeng Wu

**Affiliations:** 1 Day Ward, Binzhou People’s Hospital, Binzhou, Shandong, China; 2 The Fourth Ward of Neurology Department, Binzhou People’s Hospital, Binzhou Shandong, China; 3 The Second Ward of Pulmonary and Critical Care Medicine Department, Binzhou People’s Hospital, Binzhou, Shandong, China

**Keywords:** breast cancer-related lymphedema, lymphedema outcomes, network meta-analysis, non-pharmacological treatment, randomized controlled trials

## Abstract

**Background:**

Approximately 30% of breast cancer patients develop lymphedema after axillary surgery or radiotherapy, which impairs their quality of life and exacerbates symptoms. Non-pharmacological therapies show efficacy for breast cancer-related lymphedema (BCRL), but the optimal approach remains unclear.

**Objective:**

This study aimed to determine the most effective non-pharmacological interventions for BCRL by comparing limb volume, circumference, and pain outcomes.

**Methods:**

Cochrane, Embase, PubMed, and Web of Science databases were searched from inception to August 12, 2025, with key terms on BCRL, breast cancer, lymphedema, and non-pharmacological therapies. Cochrane RoB2 was used to assess the risk of bias. Network meta-analyses were conducted using R 4.4.1. SUCRA values were calculated to rank interventions.

**Results:**

Seventeen studies (754 patients) were included. In terms of reducing limb volume, weight reduction ranked highest (SMD: -1.2, 95% CrI [-2.1, -0.22]; SUCRA = 82.21%), followed by virtual reality (VR), exercise combined with compression therapy, and exercise therapy. For pain relief, VR-based training ranked highest (SMD: -3.7, 95% CrI [-4.5, -3.0]; SUCRA = 99.65%). However, given the sparse network topology and the limited number of included studies, these rankings should be interpreted with caution. Regarding limb circumference, the currently available evidence (which included only 3 studies with limited statistical power) did not detect any statistically significant differences among the interventions.

**Conclusions:**

Among non-pharmacological treatments for BCRL, weight reduction was most effective in improving limb volume, while VR-based training was most effective in improving pain scores in the edematous limb.

**Systematic Review Registration:**

https://www.crd.york.ac.uk/prospero/, identifier CRD420250654557.

## Introduction

Breast cancer is the most prevalent malignant tumor among women worldwide. According to the latest statistics from GLOBOCAN, there were 2.3 million new cases of breast cancer in women globally in 2020, accounting for 24.5% of all female cancer cases (1). Breast cancer-related lymphedema (BCRL) is a sequela of multimodal treatment for breast cancer. Its risk is influenced by multiple factors, and the incidence is higher among patients who undergo chemotherapy, radiotherapy, or axillary lymph node dissection (ALND), as well as those with more advanced tumor stages and higher body mass index (BMI) (2). BCRL is pathophysiologically rooted in lymphatic vessel damage caused by ALND/radiotherapy, leading to protein-rich edema that manifests as upper limb swelling, fibrosis, and functional impairment. BCRL not only significantly diminishes patients’ quality of life (with a 40% decline in quality-of-life scores) and requires lifelong management (3) but may also trigger recurrent infections and psychological distress, thereby imposing a long-term burden on health.

Current pharmacological interventions for BCRL include benzopyrones and diuretics; however, systematic reviews indicate that there is limited and low-quality evidence from relevant high-quality RCTs (4, 5). Diuretics may exacerbate interstitial fluid and electrolyte disturbances (6), and their benefits are limited in patients with concomitant fibrosis and steatosis (7, 8); antibiotics are used only for secondary infections and have high rates of resistance (9). In contrast, non-pharmacological therapies show greater promise due to their safety, feasibility, and minimal side effects. Studies have shown that complex decongestive therapy (CDT) can significantly reduce limb volume and slow the progression of fibrosis (10, 11), exercise therapy promotes lymphatic drainage through the muscle pump effect (12), and intermittent pneumatic compression therapy (CT) is also effective in reducing edema (13–15). Nevertheless, there is currently a lack of direct comparisons across interventions, treatment protocols remain unstandardized, and evidence of long-term benefits is insufficient. Therefore, this study aimed to systematically evaluate the efficacy of various non-pharmacological interventions through a network meta-analysis (NMA), to provide evidence-based guidance for the individualized management of BCRL.

In summary, pharmacological interventions are often inadequate for the long-term management of BCRL due to toxicity, dependence, and a high incidence of complications. Non-pharmacological interventions, with their advantages of high safety, greater feasibility, and controllable costs, are gradually becoming the preferred approach. However, current non-pharmacological interventions still lack sufficient evidence-based support. There is a lack of comparative studies on different interventions, standardized treatment protocols remain inconsistent, and evidence for long-term benefits in specific populations is insufficient. This study aimed to systematically evaluate evidence on non-pharmacological interventions, identify optimal clinical practice pathways, and lay the groundwork for developing personalized management strategies for BCRL.

## Materials and methods

A NMA was conducted according to the PRISMA guidelines. This NMA was registered at https://www.crd.york.ac.uk/prospero/(PROSPERO) with registration number CRD420250654557. No major modifications were made to the registered protocol during the implementation of this review.

### Search strategy

Cochrane, Embase, PubMed, and Web of Science databases were searched up to August 12, 2025. Primary search terms used included “breast cancer-related lymphedema”, “breast cancer”, “Lymphedema”, and “Non-pharmacological Therapies” (The complete search strategies for all databases, including all search terms and Boolean operators, are listed in **Supplementary Table 1**). There were no language restrictions for literature searches; however, non-English language articles must provide a necessary English abstract.

### Eligibility criteria

These eligibility criteria were developed based on the PICOS (Population, Intervention, Control, Outcome, Study design) framework:

Study population (P): Adult female patients aged 18 years or older with breast cancer confirmed by histopathological examination and clinically diagnosed with BCRL.

Interventions (I): Any non-pharmacological therapy may serve as a primary intervention, including but not limited to CDT, exercise therapy, CT, weight reduction, extracorporeal shock wave therapy (ESWT), manual lymphatic drainage (MLD), and virtual reality (VR)-based training. For the NMA, 15 distinct intervention nodes were defined; treatment approaches combined with CDT were treated as independent nodes to maintain clinical interpretability.

Comparison group (C): Standard care (typically including skin care, education, and self-management advice, but excluding intensive decongestive measures), placebo/sham intervention, or active control (e.g., the use of CDT when another intervention served as the experimental treatment).

Outcomes (O): The primary outcome measure was limb volume, the operational definitions of which were different across the included studies. Some studies reported the absolute limb volume of the affected limb, some reported excess limb volume (ELV) relative to the unaffected limb, and a few reported relative volume change or percentage of edema. All volume-related outcomes were pooled using standardized mean differences (SMD) to account for differences in measurement scales. Secondary outcomes included limb circumference and pain scores. Heterogeneity in outcome definitions was acknowledged as a limitation and further elaborated in the discussion section.

Study design (S): RCTs only.

### Literature screening

All retrieved records were imported into EndNote 21 software, and duplicate records were removed using the software’s built-in deduplication feature combined with manual verification. Two researchers independently screened titles and abstracts in EndNote 21 based on predefined inclusion and exclusion criteria; literature deemed potentially relevant was retained for full-text review. Subsequently, the same two researchers independently assessed whether the full texts met the inclusion and exclusion criteria. Any disagreements arising during any screening stage were settled through discussion or consultation with a third researcher. Patients with BCRL were the study population. Interventions comprised CDT, kinesiology taping (KT), CT, moxibustion, silicone tube implantation plus CT (silicone tube+CT), weight reduction, exercise therapy, exercise+CT, ESWT, MLD, CDT+ultrasound, CDT+faradic, CDT+fluidotherapy, CDT+CT, and VR. The control group received standard treatment (typically consisting of skin care, education, and self-management advice, but excluding intensive anti-edema components) or a placebo/sham treatment. Of note, in studies where CDT was not used as the experimental intervention, CDT itself was sometimes used as the active control, as it has been established as the standard treatment for BCRL. The primary outcome was limb volume, and secondary outcomes included limb circumference and pain scores. All studies were RCTs. During initial screening, those meeting at least the patient characteristics and intervention criteria were retained, while others were excluded.

### Inclusion and exclusion criteria

Inclusion criteria: RCTs evaluating changes in limb volume, limb circumference, and pain scores in BCRL using non-pharmacological interventions, and (1) Age ≥ 18 years (2); Patients with pathologically confirmed breast cancer and a clear diagnosis of BCRL (3); Included studies must provide appropriate outcome measures to evaluate the effects of different interventions on BCRL, such as limb volume, limb circumference, and pain scores (4); Included studies must be RCTs (5); The intervention group must receive any form of non-pharmacological therapy as the primary intervention.

Exclusion criteria (1): Studies with unclear diagnoses (2); Articles not classified as RCTs, such as reviews, conference abstracts, case reports, letters, and guidelines (3); Animal studies not involving humans (4); Studies lacking data or full-text access (5); Studies involving primary interventions with drugs (e.g., diuretics, coumarins) or surgical/invasive procedures (6); Studies reporting only baseline or intervention-period data without post-intervention outcome assessments.

### Data extraction

Two researchers reviewed and extracted data from the included articles, including first author, publication year, sample size, age, intervention, control measures, study type, follow-up period, and outcome measures (limb volume, limb circumference, pain scores).

We extracted the mean and standard deviation (SD) of changes from baseline to post-intervention. To overcome limitations associated with a single measurement unit, we used SMDs to quantify the effects of different interventions on limb volume. A sensitivity analysis stratified by outcome measure type was planned, but could not be conducted due to the limited number of studies within each category. This constituted the primary data source for the analysis in this paper. If the article did not provide the SD, calculations were performed using the standard error, 95% confidence interval (95% CI), range, and quartiles. When the interquartile range (IQR) was provided, the IQR was used as the mean, and IQR/1.135 was used as the SD. If the median of the Min-Max range appeared in eligible articles, statistical analysis of that data was excluded. Due to differences in outcome scoring scales, SMDs were used to pool data from various outcome measures.

### Risk of bias assessment

Two researchers independently assessed the risk of bias in all included RCTs using the Cochrane Collaboration’s Risk of Bias 2 (RoB 2) tool. This tool assessed five domains (1): bias in the randomization process (2); bias from deviation from the intended intervention (3); bias from missing outcome data (4); bias in outcome measurement; and (5) bias from selective reporting of results. Each domain was classified as low risk, moderate risk, or high risk using the RoB 2 algorithm. The summary plot of risk of bias was generated using Microsoft Excel. The overall quality of evidence for each outcome measure was assessed using the Grading of Recommendations, Assessment, Development, and Evaluation (GRADE) framework. Since all 17 included studies were RCTs, there was no need to use other tools for assessing risk of bias.

In this study, the GRADE approach was used to assess the quality of evidence in the NMA. For direct comparisons, evidence quality was graded based on risk of bias, inconsistency, imprecision, indirectness, and publication bias in the RCTs. For indirect comparisons, evidence quality was assessed based on the quality of the direct evidence forming the indirect evidence pathway, with particular emphasis on the validity of the transitivity assumption. When imbalanced potential effect modifiers existed across different comparisons, the level of evidence would be downgraded. Furthermore, the quality of evidence would be further downgraded if there were inconsistencies or incoherencies in the network, or if the estimates were unstable. Ultimately, the quality of evidence for the network estimate was assessed by synthesizing the quality of both direct and indirect evidence.

### Data integration and analysis

This study employed a Bayesian random-effects model for NMA, with parameter estimation based on the Markov chain Monte Carlo (MCMC) method. The model was configured with four independent Markov chains to enhance the stability and reliability of the simulation. During the iteration process, the first 25,000 iterations served as a burn-in period to eliminate the influence of initial values on the model; subsequently, 250,000 iterations were performed for parameter estimation. To reduce autocorrelation, 10-fold thinning was applied to the data, meaning that results were retained only every 10 iterations. Regarding priori distributions, non-informative priors were used to avoid introducing subjective bias into the results. For both effect sizes and heterogeneity parameters, wide distributions were adopted to reflect uncertainty. Model convergence was assessed using the Brooks–Gelman–Rubin diagnostic method, with a potential scale reduction factor (PSRF) close to 1 (typically <1.05) serving as the convergence criterion. Visual assessment was also conducted using trajectory plots and density plots. Model goodness of fit was evaluated using the Deviance Information Criterion (DIC), with lower DIC values indicating better model fit. Regarding heterogeneity, this study employed a random-effects model, assuming a certain degree of clinical and methodological differences among studies, and assessed the level of heterogeneity by estimating the between-study variance parameter (τ^2^). We compared the distribution of potential effect modifiers (such as age, baseline severity of lymphedema, type of intervention, and follow-up duration) across the studies. The results indicated no significant imbalance between comparisons, thereby supporting the validity of the transferability assumption.

To address heterogeneity within the network, we adopted the assumption of a common heterogeneity, which posits that all intervention comparisons share the same between-study standard deviation parameter τ. The square of this parameter, τ^2^, represents the between-study variance and serves as the primary quantitative measure of heterogeneity in the NMA. Since I^2^ is less direct and stable than τ^2^ in interpreting heterogeneity within the NMA, this study used it solely as a supplementary descriptive measure to help illustrate the overall level of heterogeneity. If τ^2^ equals 0, there is no heterogeneity among the studies; if τ^2^ > 0, heterogeneity exists, and a higher τ^2^ value indicates greater heterogeneity. I^2^ serves as an additional measure of heterogeneity; an I^2^ value > 50% indicates substantial heterogeneity, while an I^2^ value of 50% or less indicates that heterogeneity is within an acceptable range. If the combined results of τ^2^ and I^2^ for the included studies indicated high heterogeneity, a random-effects model was adopted; otherwise, a fixed-effects model was used. For continuous outcomes, we used either the SMD or the mean difference (MD) as the effect size, depending on the scales and units of measurement used in the original studies. For each outcome, we reported the posterior median of the effect size and its 95% credibility interval (95% CrI), along with the common heterogeneity parameter τ and its corresponding τ^2^. When only the posterior distribution of sd.d was reported, it should be interpreted as the standard deviation parameter τ across studies; the corresponding τ^2^ was obtained by squaring τ. Model fit was evaluated using residual deviance, the number of effective parameters (pD), and the deviance information criterion (DIC), where a ratio of residual deviance to the number of data points close to 1 indicated good fit.

## Results

### Study selection

**Figure 1:**
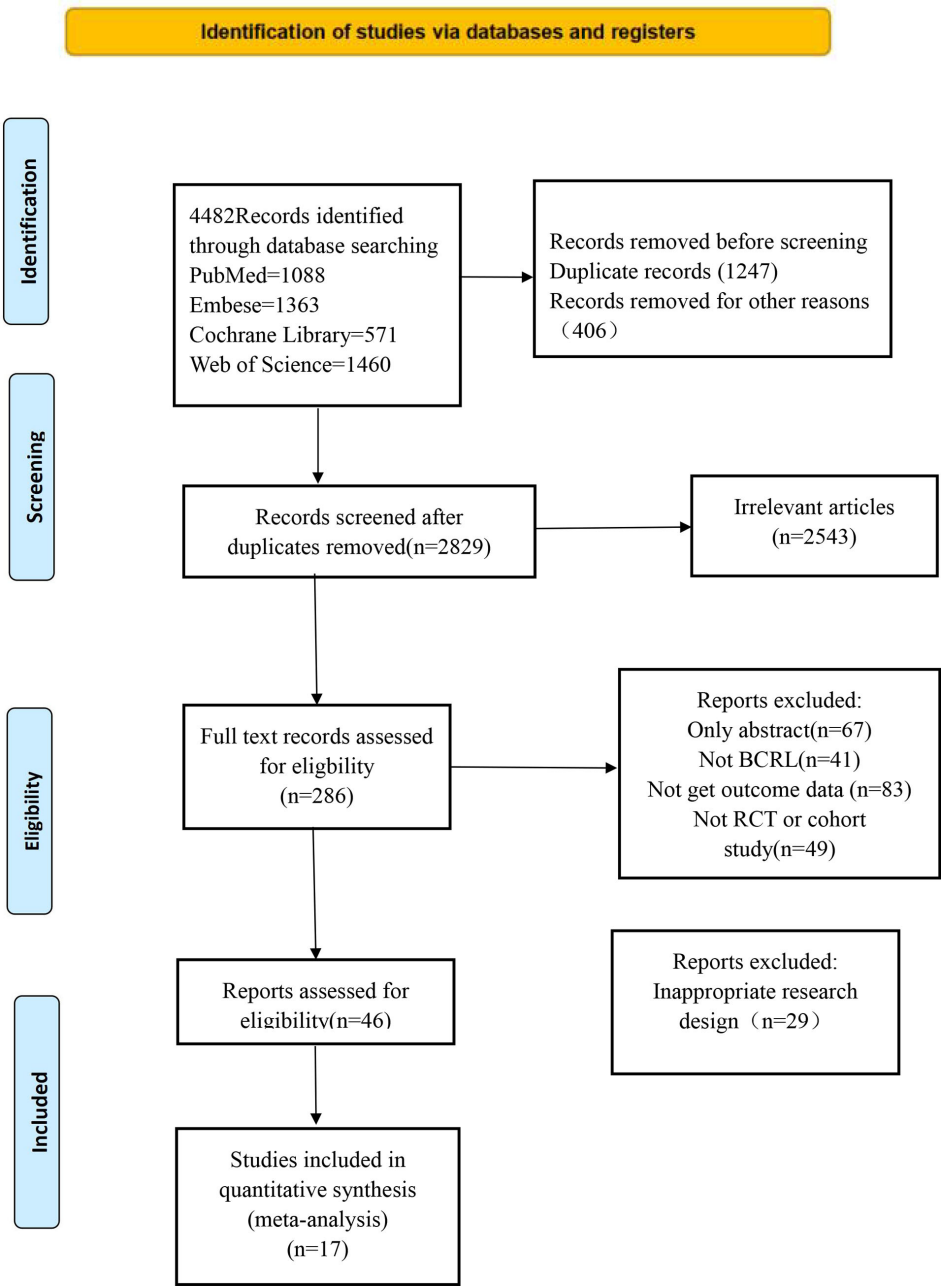
Literature screening flowchart.

A total of 4,482 articles were retrieved. After automated deduplication of 1,247 duplicates and manual deduplication of 406 duplicates, 2,543 irrelevant articles were further excluded based on the review of titles and abstracts. Among the remaining 286 articles, full texts were unavailable for 67 articles. After reviewing the full texts, 41 articles were excluded due to incompatible study populations, 83 articles due to relevant data, 49 articles due to irrelevant study type, and 29 articles due to irrational study design or incomplete data. Ultimately, 17 studies published up to August 12, 2025 were included. All 17 of these studies were RCTs; no cohort studies met the inclusion criteria. The study selection process is illustrated in the PRISMA flow diagram (**Figure 1**).

### Literature acquisition

A total of 754 patients were included across the 17 identified studies (7–23). The baseline characteristics of the included studies are summarized in **Table 1**. Fifteen interventions were reported, including CDT, KT, CT, moxibustion, Silicone Tube+CT, weight reduction, exercise therapy, exercise+CT, ESWT, MLD, CDT+Ultrasound, CDT+Faradic, CDT+Fluidotherapy, CDT+CT, and VR. The control group received either standard care or no intervention. All studies were RCTs with 2 to 24 weeks of follow-up.

**Table 1:** Baseline information.

First author	Year	Sample	Country	NCT number	Age (mean ± SD)	Intervention measures	Follow-up period	Research type	Ending
Chunhui Wang	2023	40(20/20)	China	ChiCTR1800016498	58.88 ± 6.45	TCM tuina and moxibustion VS CDT pneumatic circulation and compression garment	10 weeks	RCT	Affected limb volume, VAS scores
Mohammed T.A.Omar	2020	60(30/30)	Egypt	PACTR201802003078232	53.2 ± 2.99	Low-intensity resistance training and compression garment VS Low-intensity resistance training	12 weeks	RCT	Excess limb volume, self-reported lymphedema symptoms (Pain), DASH scores
Adarsh Pratap Singh	2020	18(8/10)	India	NA	50.57 ± 8.02	Silicone tube +CDT+BPG VS CDT+BPG	24 weeks	RCT	DASH scores, the mean volume of the limb, mean limb circumference at 10 cm above the elbow joint
Burcu Duyur Cakıt	2024	32(15/17)	Turkey	NA	60.49 ± 12.00	CDT + fluidotherapy VS CDT	3 weeks	RCT	Extremity volume, VAS scores
C. Basoglu	2021	36(19/17)	Turkey	NA	53.54 ± 8.32	CDT VS kinesiology taping (KT)	8 weeks	RCT	Limb circumference, volume, DASH scores
Clare Shaw	2007	21(11/10)	UK	NA	60	Weight reduction VS Control group	12 weeks	RCT	Limb volume
Hülya Özlem Şener	2017	60(30/30)	Turkey	NA	53.6 ± 10.33	Clinical pilates exercises VS Control group	8 weeks	RCT	VAS scores, DASH scores
Iwona Malicka	2014	28(14/14)	Poland	NA	59.8 ± 5.90	KT VS Control group	4 weeks	RCT	Limb volume
Khadra Mohamed Ali	2021	50(25/25)	Egypt	NCT04257643	50.6 ± 8.81	Aqua therapy exercises VS Land-based exercise	8 weeks	RCT	Limb volume, VAS scores
Maged A. Basha	2022	60(30/30)	Saudi Arabia	NCT04724356	50.45 ± 7.37	Xbox Kinect group VS Resistance exercise group	8 weeks	RCT	Excessive limb volume, VAS scores, DASH scores
Mehtap Aykac Cebicci	2021	20(10/10)	Turkey	NA	54.76 ± 7.32	Extracorporeal shock wave therapy VS CDT (complex decongestive therapy)	8 weeks	RCT	Volume, DASH scores
Sayed A. Tantawy	2019	59(30/29)	Egypt	NCT03401086	54.72 ± 3.74	KT group VS Pressure garment group	3 weeks	RCT	Limb circumference, pain score
A. Smykla	2013	65(20/22/23)	Poland	ACTRN12613001173785	66.38 ± 12.23	KT group, Quasi KT group VS MCT group	1 month	RCT	Percentage edema
Engin Tastaban	2020	76(38/38)	Turkey	NCT03992508	54	CDT+IPC group VS CDT group	4 weeks	RCT	Post-treatment excess volume
Mahboobeh Hemmati	2022	39(13/13/13)	Iran	IRCT201310292391N14	49.14 ± 9.99	CDT+Ultrasound group, CDT+Ultrasound group VS CDT group	2 weeks	RCT	Limb circumference at middle of the limb, volume difference
Ying Liu	2023	60(20/20/20)	China	NA	54.4 ± 9.91	MLD group, CB group VS CDT group	2 weeks	RCT	Volume
Mihriban Cagli	2025	30(15/15)	Turkey	NA	53.35 ± 10.15	CDT+Ultrasound	3 weeks	RCT	

### Risk of bias results

**Figure 2:**
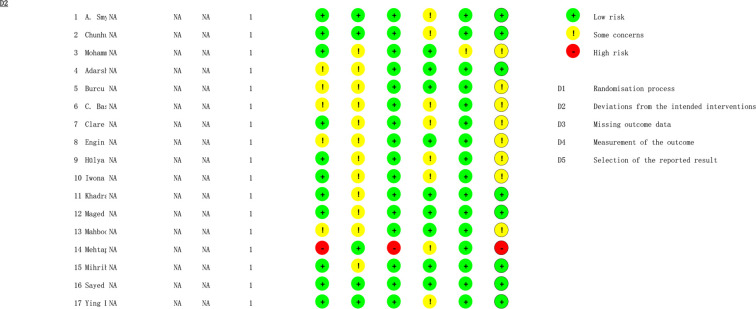
ROB2 risk assessment.

Regarding the randomization process, one study did not meet the criteria for allocation concealment and was judged as high risk; Five studies did not specify the implementation method and were judged as moderate risk. Regarding deviations from the intended interventions, 12 RCTs either did not describe blinding or failed to meet blinding requirements. However, upon further assessment, most deviations were attributed to the research environment and were unlikely to influence outcomes or interventions. Consequently, these studies were classified as having a moderate risk of bias. Regarding missing outcome data, most studies provided complete or nearly complete outcome data. Although a small proportion of studies experienced follow-up loss, the proportion was negligible and did not introduce outcome measurement bias. Therefore, these studies were assessed as low risk. One study had a missing follow-up rate of 13%, which may compromise the reliability, and was assessed as high risk. Regarding outcome measurement, eight studies failed to report their outcome measurement methods, resulting in a moderate risk assessment. Concerning selective reporting, one study did not specify a predefined analysis plan, making it impossible to assess potential bias in data analysis and reporting. This study was therefore classified as moderate risk. The detailed results are shown in **Figure 2**.

According to the GRADE evidence quality assessment criteria, the evidence level for outcomes related to limb volume in this meta-analysis was rated as moderate certainty. This was because the heterogeneity between some pairwise comparisons exceeded 50%, and one article carried a risk of downgrading due to the lack of blinding information and a high rate of loss to follow-up. Evidence certainty for pain score and limb circumference was rated as high certainty. Relevant results are presented in **Supplementary Table 2**.

### Results of heterogeneity analysis

Regarding the assessment of heterogeneity, this study employed a random-effects model for common heterogeneity in the NMA, using the common between-study variance parameter τ^2^ as the primary measure of heterogeneity and I^2^ as a supplementary descriptive indicator. For limb volume, τ = 1.03 (95% CrI 0.42–1.83), τ^2^ ≈ 1.07 (95% CrI 0.17–3.34), with an overall I^2^ of 7%. For pain, τ = 0.92 (95% CrI 0.05–2.22), τ^2^ ≈ 0.85 (95% CrI 0.00–4.95), with an overall I^2^ of 12%. For limb circumference, τ = 0.67 (95% CrI 0.03–1.36), τ^2^ ≈ 0.45 (95% CrI 0.001–1.85), with an overall I^2^ of 2%. Overall, the I^2^ values for all three outcomes suggested relatively low overall heterogeneity. However, the posterior distribution of τ/τ^2^ indicated the presence of true effect differences across studies, and the wide confidence intervals for some outcomes suggested uncertainty regarding the magnitude of heterogeneity.

### Analysis results

#### Effects of non-pharmacological therapies on limb volume

**Figure 3:**
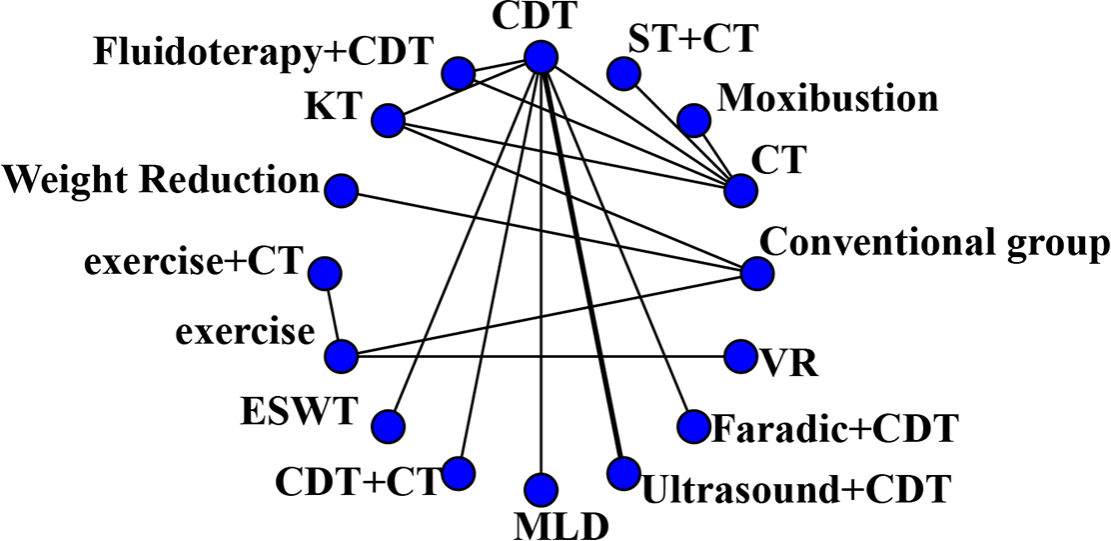
Network meta-analysis of limb volume.

**Figure 4:**
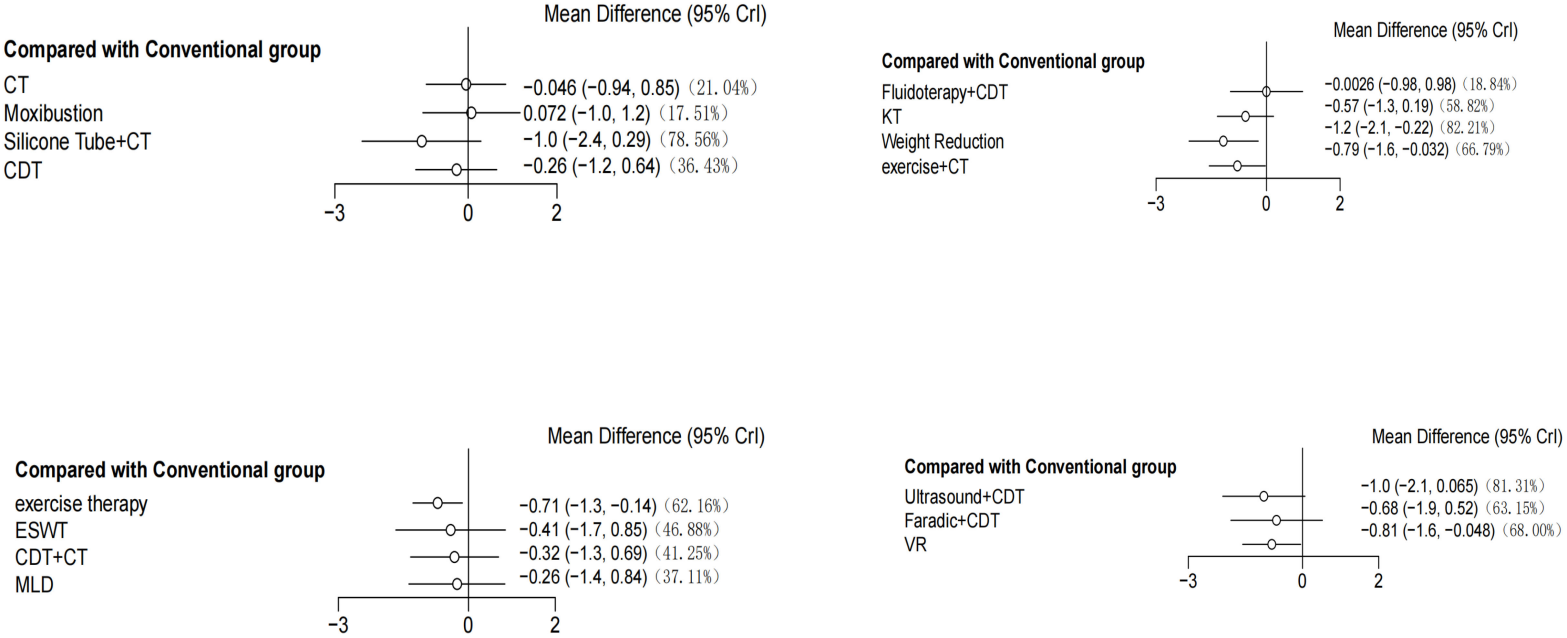
Forest plot of limb volume.

Sixteen studies (694 patients) evaluated the effects of various non-pharmacological interventions on limb volume in BCRL, including 15 interventions: CDT, KT, CT, moxibustion, Silicone Tube+CT, weight reduction, exercise therapy, exercise+CT, ESWT, MLD, CDT+Ultrasound, CDT+Faradic, CDT+Fluidotherapy, CDT+CT, and VR. The NMA results are shown in **Figure 2**. According to SUCRA scores, compared to conventional treatments, weight reduction ranked highest in terms of improving limb volume [SMD: -1.2, 95% Crl (-2.1, -0.22)] (SUCRA = 82.21%) showed significant improvement in limb volume, followed by VR [SMD: -0.81, 95% Crl (-1.6, -0.048)] (SUCRA = 68.00%), exercise+CT [SMD: -0.79, 95% Crl (-1.6, -0.032)] (SUCRA = 66.79%), and exercise therapy [SMD: -0.71, 95% Crl (-1.3, -0.14)] (SUCRA = 62.16%). Due to the lack of closed loops in the network structure, inconsistency tests for some node splitting methods could not be performed. Detailed results are presented in the NMA plot (**Figure 3**), forest plot (**Figure 4**), and league table (**Table 2**).

**Table 2:** League Table of Effects of Non-Pharmacological Interventions on Limb Volume.

Conventional group	CT	Moxibustion	ST+CT	CDT	Fluidotherapy+CDT	KT	Weight Reduction	exercise+CT	exercise	ESWT	CDT+CT	MLD	Ultrasound+CDT	Faradic+CDT	VR
**Conventional group**	**-0.05 (-0.94, 0.83)**	**0.07 (-1.02, 1.16)**	**-1.05 (-2.39, 0.29)**	**-0.26 (-1.17, 0.65)**	**0 (-0.98, 0.98)**	**-0.57 (-1.34, 0.19)**	**-1.17 (-2.11, -0.22)**	**-0.79 (-1.56, -0.03)**	**-0.71 (-1.28, -0.14)**	**-0.41 (-1.68, 0.85)**	**-0.32 (-1.34, 0.69)**	**-0.26 (-1.37, 0.84)**	**-1.02 (-2.1, 0.06)**	**-0.68 (-1.88, 0.52)**	**-0.81 (-1.57, -0.05)**
**0.05 (-0.83, 0.94)**	**CT**	**0.12 (-0.51, 0.75)**	**-1 (-2.01, 0)**	**-0.21 (-0.66, 0.23)**	**0.05 (-0.44, 0.54)**	**-0.52 (-0.98, -0.06)**	**-1.12 (-2.41, 0.17)**	**-0.74 (-1.91, 0.43)**	**-0.66 (-1.71, 0.4)**	**-0.36 (-1.34, 0.62)**	**-0.27 (-0.9, 0.36)**	**-0.21 (-0.98, 0.56)**	**-0.97 (-1.7, -0.23)**	**-0.63 (-1.53, 0.27)**	**-0.76 (-1.93, 0.42)**
**-0.07 (-1.16, 1.02)**	**-0.12 (-0.75, 0.51)**	**Moxibustion**	**-1.13 (-2.3, 0.06)**	**-0.33 (-1.11, 0.43)**	**-0.07 (-0.87, 0.72)**	**-0.64 (-1.42, 0.14)**	**-1.24 (-2.68, 0.2)**	**-0.86 (-2.18, 0.46)**	**-0.78 (-2, 0.44)**	**-0.48 (-1.65, 0.68)**	**-0.39 (-1.29, 0.49)**	**-0.34 (-1.32, 0.65)**	**-1.09 (-2.06, -0.13)**	**-0.75 (-1.85, 0.34)**	**-0.88 (-2.21, 0.45)**
1.05 (-0.29, 2.39)	**1 (0, 2.01)**	1.13 (-0.06, 2.3)	**ST+CT**	0.79 (-0.3, 1.89)	1.05 (-0.06, 2.16)	0.48 (-0.62, 1.59)	-0.12 (-1.76, 1.52)	0.26 (-1.28, 1.8)	0.34 (-1.11, 1.79)	0.64 (-0.76, 2.04)	0.73 (-0.45, 1.91)	0.79 (-0.47, 2.05)	0.03 (-1.2, 1.27)	0.37 (-0.98, 1.72)	0.25 (-1.3, 1.78)
0.26 (-0.65, 1.17)	0.21 (-0.23, 0.66)	0.33 (-0.43, 1.11)	-0.79 (-1.89, 0.3)	**CDT**	0.26 (-0.26, 0.78)	-0.31 (-0.81, 0.19)	-0.91 (-2.22, 0.4)	-0.53 (-1.71, 0.66)	-0.45 (-1.51, 0.63)	-0.15 (-1.02, 0.73)	-0.06 (-0.51, 0.39)	0 (-0.63, 0.63)	-0.75 (-1.34, -0.17)	-0.42 (-1.2, 0.37)	-0.54 (-1.73, 0.65)
0 (-0.98, 0.98)	-0.05 (-0.54, 0.44)	0.07 (-0.72, 0.87)	-1.05 (-2.16, 0.06)	-0.26 (-0.78, 0.26)	**Fluidotherapy+CDT**	-0.57 (-1.19, 0.05)	-1.17 (-2.53, 0.19)	-0.79 (-2.02, 0.46)	-0.71 (-1.84, 0.42)	-0.41 (-1.44, 0.61)	-0.32 (-1.01, 0.36)	-0.26 (-1.08, 0.56)	-1.02 (-1.8, -0.24)	-0.68 (-1.62, 0.26)	-0.81 (-2.05, 0.44)
0.57 (-0.19, 1.34)	**0.52 (0.06, 0.98)**	0.64 (-0.14, 1.42)	-0.48 (-1.59, 0.62)	0.31 (-0.19, 0.81)	0.57 (-0.05, 1.19)	**KT**	-0.6 (-1.81, 0.61)	-0.22 (-1.3, 0.86)	-0.14 (-1.09, 0.81)	0.16 (-0.85, 1.16)	0.25 (-0.42, 0.91)	0.31 (-0.49, 1.11)	-0.45 (-1.22, 0.32)	-0.11 (-1.04, 0.82)	-0.24 (-1.32, 0.84)
**1.17 (0.22, 2.11)**	1.12 (-0.17, 2.41)	1.24 (-0.2, 2.68)	0.12 (-1.52, 1.76)	0.91 (-0.4, 2.22)	1.17 (-0.19, 2.53)	0.6 (-0.61, 1.81)	**Weight Reduction**	0.38 (-0.84, 1.58)	0.46 (-0.65, 1.56)	0.76 (-0.82, 2.34)	0.85 (-0.54, 2.24)	0.91 (-0.54, 2.35)	0.15 (-1.28, 1.59)	0.49 (-1.03, 2.02)	0.36 (-0.85, 1.57)
**0.79 (0.03, 1.56)**	0.74 (-0.43, 1.91)	0.86 (-0.46, 2.18)	-0.26 (-1.8, 1.28)	0.53 (-0.66, 1.71)	0.79 (-0.46, 2.02)	0.22 (-0.86, 1.3)	-0.38 (-1.58, 0.84)	**exercise+CT**	0.08 (-0.43, 0.59)	0.38 (-1.1, 1.85)	0.47 (-0.8, 1.75)	0.53 (-0.82, 1.87)	-0.23 (-1.55, 1.09)	0.11 (-1.32, 1.53)	-0.02 (-0.73, 0.7)
**0.71 (0.14, 1.28)**	0.66 (-0.4, 1.71)	0.78 (-0.44, 2)	-0.34 (-1.79, 1.11)	0.45 (-0.63, 1.51)	0.71 (-0.42, 1.84)	0.14 (-0.81, 1.09)	-0.46 (-1.56, 0.65)	-0.08 (-0.59, 0.43)	**exercise**	0.3 (-1.09, 1.68)	0.39 (-0.77, 1.55)	0.45 (-0.79, 1.68)	-0.31 (-1.53, 0.91)	0.03 (-1.31, 1.36)	-0.1 (-0.61, 0.41)
0.41 (-0.85, 1.68)	0.36 (-0.62, 1.34)	0.48 (-0.68, 1.65)	-0.64 (-2.04, 0.76)	0.15 (-0.73, 1.02)	0.41 (-0.61, 1.44)	-0.16 (-1.16, 0.85)	-0.76 (-2.34, 0.82)	-0.38 (-1.85, 1.1)	-0.3 (-1.68, 1.09)	**ESWT**	0.09 (-0.9, 1.07)	0.15 (-0.93, 1.22)	-0.6 (-1.66, 0.45)	-0.27 (-1.45, 0.91)	-0.4 (-1.88, 1.08)
0.32 (-0.69, 1.34)	0.27 (-0.36, 0.9)	0.39 (-0.49, 1.29)	-0.73 (-1.91, 0.45)	0.06 (-0.39, 0.51)	0.32 (-0.36, 1.01)	-0.25 (-0.91, 0.42)	-0.85 (-2.24, 0.54)	-0.47 (-1.75, 0.8)	-0.39 (-1.55, 0.77)	-0.09 (-1.07, 0.9)	**CDT+CT**	0.06 (-0.71, 0.83)	-0.7 (-1.43, 0.04)	-0.36 (-1.26, 0.55)	-0.49 (-1.76, 0.78)
0.26 (-0.84, 1.37)	0.21 (-0.56, 0.98)	0.34 (-0.65, 1.32)	-0.79 (-2.05, 0.47)	0 (-0.63, 0.63)	0.26 (-0.56, 1.08)	-0.31 (-1.11, 0.49)	-0.91 (-2.35, 0.54)	-0.53 (-1.87, 0.82)	-0.45 (-1.68, 0.79)	-0.15 (-1.22, 0.93)	-0.06 (-0.83, 0.71)	**MLD**	-0.75 (-1.61, 0.09)	-0.42 (-1.43, 0.59)	-0.55 (-1.88, 0.8)
1.02 (-0.06, 2.1)	**0.97 (0.23, 1.7)**	**1.09 (0.13, 2.06)**	-0.03 (-1.27, 1.2)	**0.75 (0.17, 1.34)**	**1.02 (0.24, 1.8)**	0.45 (-0.32, 1.22)	-0.15 (-1.59, 1.28)	0.23 (-1.09, 1.55)	0.31 (-0.91, 1.53)	0.6 (-0.45, 1.66)	0.7 (-0.04, 1.43)	0.75 (-0.09, 1.61)	**Ultrasound+CDT**	0.34 (-0.64, 1.32)	0.21 (-1.12, 1.53)
0.68 (-0.52, 1.88)	0.63 (-0.27, 1.53)	0.75 (-0.34, 1.85)	-0.37 (-1.72, 0.98)	0.42 (-0.37, 1.2)	0.68 (-0.26, 1.62)	0.11 (-0.82, 1.04)	-0.49 (-2.02, 1.03)	-0.11 (-1.53, 1.32)	-0.03 (-1.36, 1.31)	0.27 (-0.91, 1.45)	0.36 (-0.55, 1.26)	0.42 (-0.59, 1.43)	-0.34 (-1.32, 0.64)	**Faradic+CDT**	-0.12 (-1.55, 1.3)
**0.81 (0.05, 1.57)**	0.76 (-0.42, 1.93)	0.88 (-0.45, 2.21)	-0.25 (-1.78, 1.3)	0.54 (-0.65, 1.73)	0.81 (-0.44, 2.05)	0.24 (-0.84, 1.32)	-0.36 (-1.57, 0.85)	0.02 (-0.7, 0.73)	0.1 (-0.41, 0.61)	0.4 (-1.08, 1.88)	0.49 (-0.78, 1.76)	0.55 (-0.8, 1.88)	-0.21 (-1.53, 1.12)	0.12 (-1.3, 1.55)	**VR**

#### Effects of non-pharmacological therapies on limb circumference

**Figure 5:**
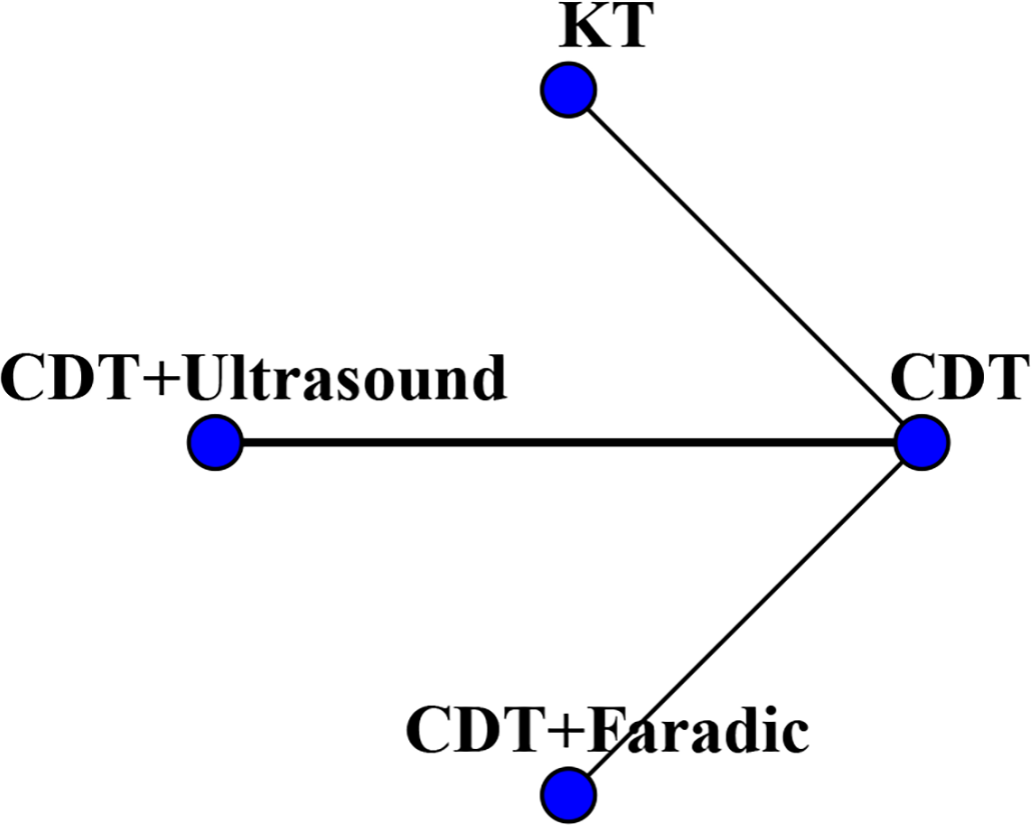
Network meta-analysis of limb circumference.

**Figure 6:**
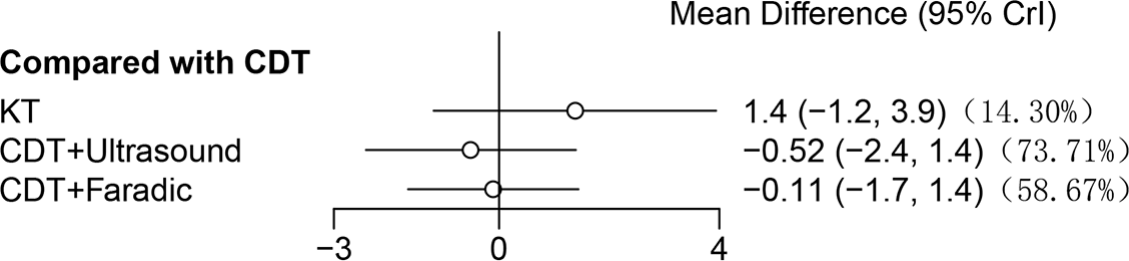
Forest plot of limb circumference.

Three studies (105 patients) evaluated the effects of different non-pharmacological interventions on limb circumference in BCRL patients, including 4 interventions: CDT, KT, CDT+Ultrasound, and CDT+Faradic. NMA results are shown in **Figure 5**. Based on the forest plot of relative effects and the league table, no statistically significant differences were observed among interventions in terms of limb circumference. However, given that the network for this outcome included only three studies and had sparse connections, statistical power was limited; therefore, this negative finding should be interpreted with caution. Due to the lack of closed loops in the network structure, inconsistency tests for some node splitting methods could not be performed. Similarly, homogeneity tests could not be conducted because the network structure failed to form closed loops. Detailed results are presented in the NMA plot (**Figure 5**), forest plot (**Figure 6**), and league table (**Table 3**).

**Table 3:** League Table of Effects of Non-Pharmacological Interventions on Limb Circumference.

CDT	KT	CDT+Ultrasound	CDT+Faradic
**CDT**	**1.38 (-1.19, 3.94)**	**-0.52 (-2.42, 1.39)**	**-0.11 (-1.65, 1.44)**
-1.38 (-3.94, 1.19)	**KT**	-1.9 (-5.1, 1.3)	-1.48 (-4.47, 1.48)
0.52 (-1.39, 2.42)	1.9 (-1.3, 5.1)	**CDT+Ultrasound**	0.42 (-2.04, 2.86)
0.11 (-1.44, 1.65)	1.48 (-1.48, 4.47)	-0.42 (-2.86, 2.04)	**CDT+Faradic**

#### Effects of non-pharmacological interventions on pain scores

**Figure 7:**
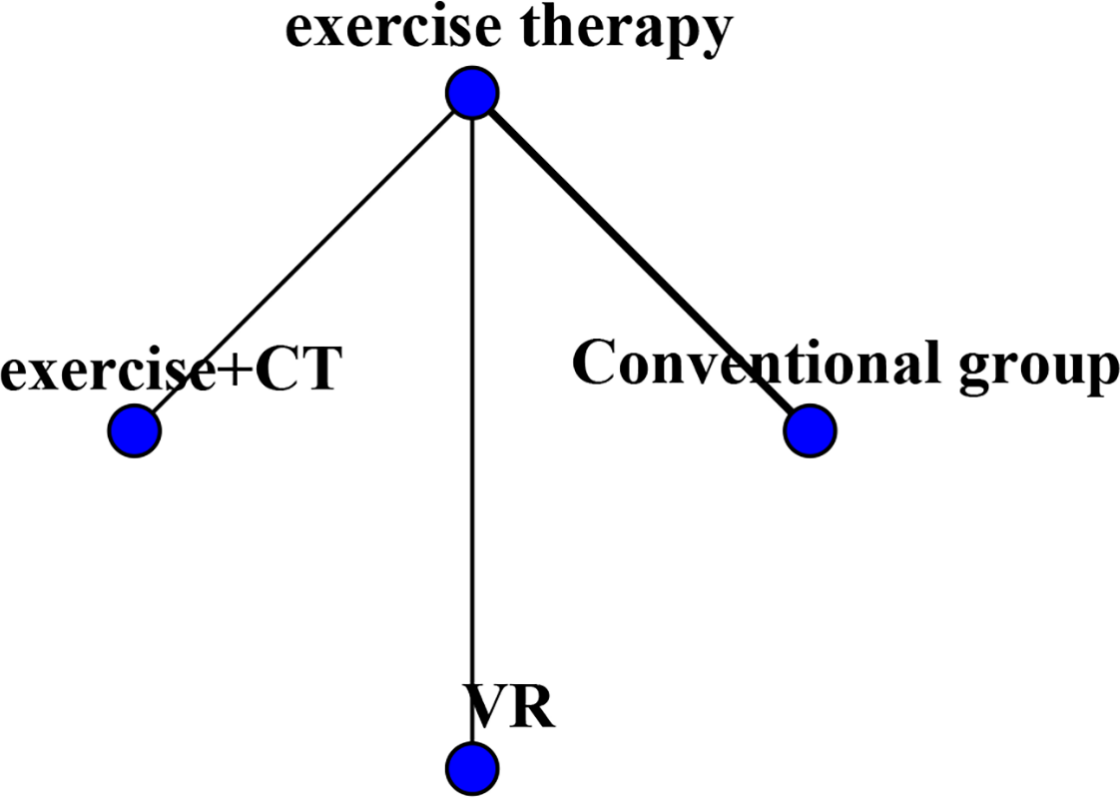
Network meta-analysis of pain.

**Figure 8:**
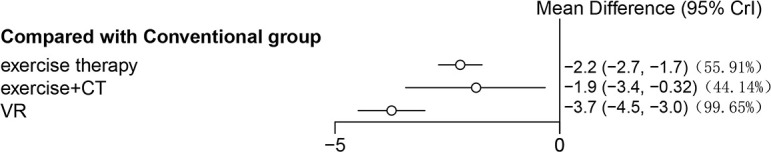
Forest plot of pain.

Four studies (230 patients) evaluated the impact of various non-pharmacological interventions on pain scores, including 3 interventions: exercise therapy, exercise+CT, and VR-based training. NMA results are presented in **Figure 7**. According to the SUCRA score, VR-based training demonstrated the most significant effect in reducing pain in the edematous limb compared to standard treatment [SMD: -3.7, 95% Crl (-4.5, -3.0)] (SUCRA = 99.65%), followed by exercise therapy [SMD: -2.2, 95% Crl (-2.7, -1.7)] (SUCRA = 55.91%) and exercise+CT [SMD: -1.9, 95% Crl (-3.4, -0.32)] (SUCRA = 44.14%). Due to the lack of closed loops in the network structure, inconsistency tests for some node splitting methods could not be performed. Similarly, homogeneity tests could not be conducted because the network structure failed to form closed loops. Detailed results are presented in the NMA plot (**Figure 7**), forest plot (**Figure 8**), and league table (**Table 4**).

**Table 4:** League Table of Effects of Non-Pharmacological Interventions on Pain Scores.

Conventional group	exercise therapy	exercise+CT	VR
Conventional group	**-2.21 (-2.7, -1.73)**	**-1.86 (-3.43, -0.32)**	**-3.74 (-4.49, -3)**
2.21 (1.73, 2.7)	**exercise therapy**	0.35 (-1.15, 1.82)	-1.53 (-2.09, -0.96)
1.86 (0.32, 3.43)	-0.35 (-1.82, 1.15)	**exercise+CT**	-1.88 (-3.45, -0.28)
3.74 (3, 4.49)	**1.53 (0.96, 2.09)**	**1.88 (0.28, 3.45)**	**VR**

## Discussion

This NMA synthesized data from 17 RCTs involving 754 patients with BCRL. It evaluated the comparative effectiveness of various non-pharmacological interventions on limb volume, limb circumference, and pain scores. The results revealed that, based on SUCRA ranking probabilities, weight reduction ranked highest in improving limb volume, followed by VR-based training, exercise + CT, and exercise therapy. Regarding pain relief, VR-based training ranked highest. However, given the sparse network topology, particularly the fact that the pain outcome included only three interventions and lacked a closed loop, these rankings should be interpreted as exploratory and hypothesis-generating rather than as definitive evidence of superiority. The probability-based nature of the SUCRA ranking implies that interventions with higher SUCRA values are more likely to be effective; however, this does not establish their clinical superiority without confirmation from larger head-to-head trials. Regarding limb circumference, no statistically significant differences were detected among the interventions evaluated. This finding may reflect the limited number of included studies (n=3) and the resulting lack of statistical power, rather than providing definitive evidence of ineffectiveness. Future studies with larger sample sizes are needed to fully evaluate the comparative effectiveness of non-pharmacological interventions on this outcome.

In analyzing improvements in limb volume, we examined various metrics, including affected limb volume and ELV. A NMA ranked weight reduction as the top-ranked intervention for improving limb volume. Research has demonstrated a significant correlation between weight reduction and limb volume reduction (14), suggesting a potential association worthy of further investigation. However, the specific physiological mechanisms underlying the relationship between weight reduction and lymphedema improvement, whether through reduced adipose tissue, decreased inflammatory burden, or improved lymphatic function, were not directly assessed in the included studies and remain to be elucidated in future research. Research indicates that VR can enhance adherence to self-management programs by increasing patient engagement and motivation. Specifically, VR-based exercise games are viewed as viable weight control options by younger patients due to their enjoyable and convenient characteristics. Other studies have shown that wearable VR technology holds promise for clinical applications in the long-term management of BCRL. Particularly in telemedicine settings, portable 3D imaging tools have been demonstrated to accurately assess BCRL in both clinical and home environments (24). Furthermore, VR can visually present complex medical information, helping cancer patients better understand their condition and treatment plans (25). This educational function may indirectly enhance patients’ understanding of vascular tension-related therapies and enhance treatment adherence. The plausible mechanisms proposed in the literature suggest that exercise may enhance lymphatic flow through mechanical compression, molecular signaling, and metabolic regulation. It is believed that structured resistance training promotes lymphatic drainage through the pumping action of muscle contractions, thereby reducing the accumulation of interstitial fluid. Evidence indicates that resistance training does not exacerbate lymphedema; rather, it improves fluid balance in the upper limbs and increases lean body mass (28). Similarly, it has been proposed that CT modulates the tissue microenvironment through external mechanical intervention; compression sleeves or bandages may increase tissue hydrostatic pressure via external pressure, thereby promoting concentric lymphatic drainage and reducing protein and fluid retention (29, 30). Long-term CT may also inhibit fibrosis and adipose tissue proliferation, thereby maintaining tissue compressibility (31). Although these mechanisms are biologically plausible and supported by preclinical and observational studies, they have not been directly validated in the included RCTs and should be regarded as explanatory hypotheses rather than established causal pathways derived from this NMA. Exercise (such as resistance training) promotes lymphatic drainage through active muscle pumping, while CT provides passive support. Combining both approaches may be more effective in maintaining fluid balance (26–28). In clinical practice, weight reduction programs should be prioritized for BCRL patients. Additionally, VR-based technology can be utilized to assist in nursing care, thereby improving patients’ adherence and disease perception. Furthermore, patients should be guided to perform structured resistance training combined with CT to facilitate precision care and improve limb volume.

Regarding improvements in limb circumference, no effective interventions were identified through analysis. This may be attributed to the limited number of included studies. Although this analysis did not identify any effective interventions, the underlying reason lies in the limited statistical power resulting from insufficient literature rather than the ineffectiveness of interventions themselves. Future research could address this by expanding search scopes to increase the number of available studies.

In terms of pain relief, VR-based training ranked highest in the SUCRA analysis, followed by exercise therapy and exercise + CT. The analgesic effects of VR observed in this analysis are consistent with findings in the broader literature on pain, where VR can reduce pain intensity by diverting attention and improving mood (34–36, 38). However, it should be noted that the included VR trials did not measure attention or emotional mediators; therefore, the mechanisms underlying the observed pain reduction remain speculative and should be interpreted as biologically plausible hypotheses supported by external evidence, rather than direct conclusions of this NMA. Future studies incorporating formal mediation analyses would help elucidate the specific pathways through which VR exerts its analgesic effects in patients with BCRL. In clinical practice, VR may be prioritized to alleviate pain in BCRL patients. Its immersive environment can divert attention and improve mood while guiding patients through exercises or CT. Concurrently, the long-term efficacy and immediate effects of analgesic interventions should be leveraged to enhance pain management quality.

This NMA simultaneously compared 15 distinct non-pharmacological interventions, covering three core indicators: limb volume, limb circumference, and pain. It also incorporated emerging technologies such as VR and ultrasound, thereby providing cutting-edge, feasible solutions for clinical practice. However, it should be noted that some RCTs did not provide detailed randomization or blinding procedures, which may have led to an overestimation of treatment efficacy. Furthermore, no effective interventions were identified for improving limb circumference, which may be attributed to the limited number of included studies. Third, because patient-level characteristics (such as ISL staging and baseline severity) were not fully reported in the included studies, it was not possible to conduct subgroup analyses to investigate whether treatment outcomes differed across different patient subgroups. Therefore, these results should be interpreted as comparisons of overall efficacy rather than as evidence supporting personalized treatment selection. Fourth, there is considerable heterogeneity in the operational definitions of the primary outcome measures across the included studies. While all studies assessed some aspect of limb volume, the specific measures varied, including absolute limb volume, ELV relative to the unaffected limb, and percentage of edema. Although the SMD allows for the statistical pooling of outcomes measured on different scales, these measures may reflect different biological constructs. For example, absolute limb volume is influenced by systemic factors, such as weight gain, whereas ELV specifically captures changes attributable to lymphedema. This heterogeneity should be considered when interpreting comparative effect estimates. Fifth, the evidence network structure in this study has certain limitations. Primarily, the network is relatively sparse overall, and there is a lack of direct comparisons between some interventions. Secondly, most intervention comparisons do not form a closed-loop structure, thereby limiting the formal testing of consistency between direct and indirect evidence. Furthermore, the distribution of included studies varies significantly across different interventions; some interventions are supported by only a small number of studies, which may affect the robustness of the ranking results. Therefore, caution should be exercised when interpreting the study results, particularly for interventions with limited evidence. Sixth, and importantly, because the included studies did not adequately report key patient-level characteristics, the assumption of generalizability upon which this NMA relies cannot be formally validated. Important potential effect modifiers, including baseline ISL stage, BMI, postoperative time, and fibrosis burden, were reported inconsistently or were entirely missing across studies. This heterogeneity in the study populations, combined with the diverse mechanisms of action of the included interventions (ranging from physical modalities, such as exercise and CT, to weight loss and VR rehabilitation), casts doubt on the validity of certain indirect comparisons. Readers should exercise appropriate caution when interpreting estimates of comparative effects, particularly those derived solely from indirect evidence. Seventh, it should be noted that although the overall I^2^ values for the three outcomes were low, within the framework of a NMA, heterogeneity should primarily be interpreted based on the variance parameter τ^2^ across studies. In particular, for limb volume and pain, the confidence intervals for τ^2^ were wide, suggesting uncertainty in the magnitude of heterogeneity. Therefore, the estimated network effects and SUCRA rankings should be interpreted with caution.

## Conclusion

This NMA suggests that, among the non-pharmacological interventions evaluated, weight reduction ranked highest in terms of improving limb volume, followed by VR-based training, exercise + CT, and exercise therapy. In terms of pain relief, VR-based training ranked highest. However, these findings stem from sparse evidence, a limited number of studies, and few direct comparisons; therefore, they should be regarded as exploratory findings generating hypotheses and require confirmation in larger, well-designed head-to-head RCTs. Within the current evidence (limited to only three studies with sparse network connections), no statistically significant differences in limb circumference were detected; therefore, definitive conclusions cannot be drawn regarding this outcome.

## Data Availability

The original contributions presented in the study are included in the article/**Supplementary Material**. Further inquiries can be directed to the corresponding author.
