# Supplementary material for: Non-pharmacological therapies for breast cancer-related lymphedema: a systematic review and network meta-analysis based on randomized controlled trials
Source: Front Oncol. 2026 May 29;16:1828957. doi: 10.3389/fonc.2026.1828957 (PMC13259749; doi:10.3389/fonc.2026.1828957)
Supplement: Supplementary file 1 [file Table1.docx]

**Supplementary Materials**

**Supplementary Table S1 Search Results**

| Pubmed | | |
| --- | --- | --- |
| # | Query | Results |
| 1 | "Breast Cancer Lymphedema"[Mesh] | 3670 |
| 2 | breast cancer lymphoedema*[Title/Abstract] OR Breast Cancer Related Arm Lymphedema[Title/Abstract] OR Breast Cancer Related Lymphedema[Title/Abstract] OR Breast Cancer Treatment Related Lymphedema[Title/Abstract] OR breast cancer-related lymp[Title/Abstract] OR breast cancer-related lymphoedema[Title/Abstract] OR post-breast cancer lymphedema[Title/Abstract] OR post-breast cancer lymphoedema[Title/Abstract] OR Postmastectomy Lymphedemas[Title/Abstract] OR Post-mastectomy Lymphedemas[Title/Abstract] OR BCRL[Title/Abstract] | 1366 |
| 3 | #1 OR #2 | 1478 |
| 4 | Breast Neoplasms[Title/Abstract] OR Breast cancer[Title/Abstract] OR Breast tumor[Title/Abstract] | 394821 |
| 5 | Lymphedema[Title/Abstract] | 11704 |
| 6 | #4 AND #5 | 3057 |
| 7 | #3 OR #6 | 3263 |
| 8 | "randomized controlled trial"[Title/Abstract] OR "controlled clinical trial"[Title/Abstract] OR "randomized"[Title/Abstract] OR "randomised"[Title/Abstract] OR "placebo"[Title/Abstract] OR "drug therapy"[Title/Abstract] OR "randomly"[Title/Abstract] OR "trial"[Title/Abstract] OR "groups"[Title/Abstract] NOT "animals"[Title/Abstract] | 4021484 |
| 9 | #7 and #8 | 1088 |

| Embase | | |
| --- | --- | --- |
| # | Query | Results |
| 1 | 'breast cancer-related lymphedema'/exp | 1194 |
| 2 | 'breast cancer lymphoedema*':ab,ti OR 'breast cancer related arm lymphedema':ab,ti OR 'breast cancer related lymphedema':ab,ti OR 'breast cancer treatment related lymphedema':ab,ti OR 'breast cancer-related lymp':ab,ti OR 'breast cancer-related lymphoedema':ab,ti OR 'post-breast cancer lymphedema':ab,ti OR 'post-breast cancer lymphoedema':ab,ti OR 'postmastectomy lymphedemas':ab,ti OR 'post-mastectomy lymphedemas':ab,ti OR bcrl:ab,ti | 1606 |
| 3 | #1 OR #2 | 1879 |
| 4 | 'breast neoplasms':ab,ti OR 'breast cancer':ab,ti OR 'breast tumor':ab,ti | 534482 |
| 5 | lymphedema:ab,ti | 15208 |
| 6 | #4 AND #5 | 4139 |
| 7 | ('randomized controlled trial':ab,ti OR 'controlled clinical trial':ab,ti OR 'randomized':ab,ti OR 'randomised':ab,ti OR 'placebo':ab,ti OR 'drug therapy':ab,ti OR 'randomly':ab,ti OR 'trial':ab,ti OR 'groups':ab,ti) NOT animals:ab,ti | 5373035 |
| 8 | #3 OR #6 | 4523 |
| 9 | #7 AND #8 | 1363 |

| Cochrane Library | | |
| --- | --- | --- |
| # | Query | Results |
| 1 | MeSH descriptor: [Breast Cancer Lymphedema] explode all trees | 173 |
| 2 | (breast cancer lymphoedema* OR Breast Cancer Related Arm Lymphedema OR Breast Cancer Related Lymphedema OR Breast Cancer Treatment Related Lymphedema OR breast cancer-related lymp OR breast cancer-related lymphoedema OR post-breast cancer lymphedema OR post-breast cancer lymphoedema OR Postmastectomy Lymphedemas OR Post-mastectomy Lymphedemas OR BCRL):ti,ab,kw | 749 |
| 3 | #1 or #2 | 782 |
| 4 | (Breast Neoplasms OR Breast cancer OR Breast tumor):ti,ab,kw | 49486 |
| 5 | (Lymphedema):ti,ab,kw | 2036 |
| 6 | #4 and #5 | 1242 |
| 7 | #3 and #6 | 777 |
| 8 | (“randomized controlled trial” OR “controlled clinical trial” OR “randomized” OR “randomised” OR “placebo” OR “drug therapy” OR “randomly” OR “trial” OR “groups” NOT ”animals“):ti,ab,kw | 1697692 |
| 9 | #7 and #8 | 571 |

| Web of science | | |
| --- | --- | --- |
| # | Query | Results |
| 1 | TS=(Breast Cancer Lymphedema) | 4417 |
| 2 | TS=(breast cancer lymphoedema* OR Breast Cancer Related Arm Lymphedema OR Breast Cancer Related Lymphedema OR Breast Cancer Treatment Related Lymphedema OR breast cancer-related lymp OR breast cancer-related lymphoedema OR post-breast cancer lymphedema OR post-breast cancer lymphoedema OR Postmastectomy Lymphedemas OR Post-mastectomy Lymphedemas OR BCRL) | 2722 |
| 3 | #1 OR #2 | 4814 |
| 4 | TS=(Breast Neoplasms OR Breast cancer OR Breast tumor) | 688320 |
| 5 | TS=(Lymphedema) | 11445 |
| 6 | #4 AND #5 | 4448 |
| 7 | #3 AND #6 | 4417 |
| 8 | TS=(“randomized controlled trial” OR “controlled clinical trial” OR “randomized” OR “randomised” OR “placebo” OR “drug therapy” OR “randomly” OR “trial” OR “groups” NOT ”animals“) | 4977057 |
| 9 | #7 AND #8 | 1460 |

**Supplementary Table S2** Grade Evidence Quality Assessment

| Certainty assessment | | | | | | | Certainty |
| --- | --- | --- | --- | --- | --- | --- | --- |
| № of studies | Study design | Risk of bias | Inconsistency | Indirectness | Imprecision | Other considerations |  |
| Body volume | | | | | | | |
| 16 | randomised trials | serious^a^ | serious^b^ | not serious | not serious | none | ⨁⨁◯◯ Moderate^a,b^ |
| Pain score | | | | | | | |
| 4 | randomised trials | not serious | not serious | not serious | not serious | none | ⨁⨁⨁⨁ High |
| Arm circumference | | | | | | | |
| 3 | randomised trials | not serious | not serious | not serious | not serious | none | ⨁⨁⨁⨁ High |

CI: confidence interval

Explanations

a. The allocation sequence is not described as random.

b. Study heterogeneity I^2^=52%
